# Hepatitis A, B, and A/B vaccination series completion among US adults: A claims-based analysis

**DOI:** 10.1080/21645515.2018.1489189

**Published:** 2018-07-12

**Authors:** Parinaz K. Ghaswalla, Brandon J. Patterson, Wendy Y. Cheng, Emilie Duchesneau, Monica Macheca, Mei Sheng Duh

**Affiliations:** a GSK, Philadelphia, PA, USA; b Analysis Group, Inc., Boston, MA, USA

**Keywords:** adult, hepatitis A vaccination, hepatitis B vaccination, immunization schedule, United States

## Abstract

Hepatitis A and B disease burden persists in the US. We assessed hepatitis A and hepatitis B vaccination series completion rates among 350,240 commercial/Medicare and 12,599 Medicaid enrollees aged ≥19 years. A vaccination series was considered as completed provided that the minimum interval between doses, as defined by the CDC, and the minimum number of doses were reached. We stratified completion rates by vaccine type (i.e. monovalent or bivalent) at initial vaccination for each cohort. In the commercial/Medicare cohort, the series completion rate was 32.0% for hepatitis A and 39.6% for hepatitis B among those who initiated with a monovalent vaccine, and it was 36.2% for hepatitis A and 48.9% for hepatitis B among those who initiated with a bivalent vaccine. In the Medicaid cohort, the series completion rate was 21.0% for hepatitis A and 24.0% for hepatitis B among those who initiated with a monovalent vaccine, and it was 19.0% for hepatitis A and 24.6% for hepatitis B among those who initiated with a bivalent vaccine. In conclusion, hepatitis A and B vaccination series completion rates were low, and appeared to be lower among Medicaid than among commercial/Medicare enrollees. Commercial/Medicare enrollees who initiated with a bivalent vaccine had higher series completion rates than those who initiated with monovalent vaccines – an observation that was not made among Medicaid enrollees.

## FOCUS ON THE PATIENT

*What is the context?*

- Hepatitis A and B are vaccine-preventable liver diseases. Although the reported incidence of hepatitis A and hepatitis B infections have dropped significantly since the introduction of hepatitis vaccines, surveillance reports indicate that hepatitis A and B infections persist in adults.
- There is limited information on hepatitis A and hepatitis B vaccine series completion.

*What is new?*

- In our large cohort study we found that commercial/Medicare and Medicaid enrollees had low rates of vaccination series completion for both hepatitis A and hepatitis B vaccines.

*What is the impact?*

- These results demonstrate the need for interventions to increase hepatitis A and B vaccine series completion.

## Introduction

Hepatitis A and B are vaccine-preventable liver diseases caused by hepatitis A (HAV) and hepatitis B viruses (HBV), respectively.^1^^,^^2^ Since the Centers for Disease Control and Prevention's (CDC) introduction of hepatitis A vaccination recommendations for infants in high-risk communities in 1996,^3^ followed by a recommendation for routine pediatric vaccination nationwide in 2006,^4^ and of routine hepatitis B vaccination recommendations for children in 1991,^5^ the reported incidence of hepatitis A has dropped by more than 90% and that of acute hepatitis B by more than 80% in the United States (US).^6^

Despite this decrease, the disease burden of hepatitis A and B persists. Consistently, the US National Viral Hepatitis Action Plan 2017–2020 states that hepatitis B is still a serious threat to the health of the US population despite a ‘coordinated national response’ initiated in 2011^7^. In 2015, the estimated number of new HAV infections in the US was 2,800 (95% confidence interval [CI] = 1,900–3,100), while that for new HBV infections was 21,900 (95% CI = 12,500–53,600), representing increases over the previous year of 12% and 14%, respectively.^8^^,^^9^

For adults at high risk for infection, morbidity, or mortality, the US Advisory Committee on Immunization Practices (ACIP) issued specific recommendations for hepatitis A^4^ and hepatitis B vaccinations.^10^ In the US, the available types of hepatitis vaccines for adults are the monovalent hepatitis A (HepA) vaccines, the monovalent hepatitis B (HepB) vaccines, or a combined bivalent hepatitis A/hepatitis B (HepA-HepB) vaccine.^11^^,^^12^ The monovalent HepA vaccines are inactivated whole-virus vaccines, and the monovalent HepB vaccines are recombinant vaccines. The bivalent HepA-HepB vaccine contains one pediatric dose of monovalent HepA vaccine and one adult dose of monovalent HepB vaccine.^11^^,^^12^ Potential reasons for the persisting vaccine-preventable hepatitis disease burden may be suboptimal vaccination rates and poor vaccination coverage among adults, especially among adults at a high-risk for hepatitis A or B, compared to infants and children.^7^^,^^13^ In 2015, for adults aged ≥19 years, reported HepA vaccination coverage (≥2 doses) and HepB vaccination coverage (≥3 doses) was 9.0% and 24.6%, respectively.^13^

Literature on adherence to the CDC guidelines for HepA, HepB, and HepA-HepB vaccine series completion schedules among adults in the US is limited. One study^14^ determined that HepA and HepB series completion rates in the Vaccine Safety Datalink population ranged between 40% to 65% among those who received a first dose, depending on the hepatitis vaccine (HepA or HepB), with higher completion rates in those aged <18 years. However, this study was done prior to the US Food and Drug Administration approval of bivalent vaccine in 2001.^15^ Bivalent vaccine introduction was expected to increase adherence to vaccination schedule, by offering fewer medical visits and by reducing the number of injections required by the separate administration of each monovalent vaccine.^16^ Other studies assessing HepA, HepB or HepA-HepB series completion were either based on a select patient population, such as travelers,^17^ or were conducted outside the US.^17^^,^^18^

In this context, we conducted a retrospective longitudinal cohort study using recent insurance claims data to assess HepA and HepB vaccination series completion rates among US adult enrollees of commercial/Medicare and Medicaid insurances, separately. We further stratified the results by type of vaccine, monovalent or bivalent, that was received when the vaccination series was initiated.

## Results

The eligible population consisted of 350,240 commercial/Medicare patients and 12,599 Medicaid patients. Table 1 summarizes patient demographics based on vaccination series; mean age ranged from 42.1 to 45.8 years among commercial/Medicare patients and 37.1 to 40.2 years among Medicaid patients; over half of commercial/Medicare patients (55.3-60.0%) were female and most Medicaid patients (66.9-75.1%) were female.

**Table 1.** Baseline characteristics of commercial/Medicare and of Medicaid enrollees who meet study selection criteria, by type of vaccine at initial vaccination dose.

	Commercial/Medicare			Medicaid		
	Hepatitis A vaccine	Hepatitis B vaccine	Hepatitis A/B vaccine	Hepatitis A vaccine	Hepatitis B vaccine	Hepatitis A/B vaccine
	N = 183,000	N = 147,453	N = 64,870	N = 3,570	N = 7,189	N = 3,063
Age, mean (SD)	42.1 (14.8)	44.4 (14.2)	45.8 (11.6)	37.1 (13.2)	38.9 (12.3)	40.2 (11.9)
Female, %	55.3	55.4	60	66.9	75.1	70.9
Health plan type, %						
PPO	55.7	56.5	49.8	NA	NA	NA
HMO	21.1	19.0	18.2	41.1	49.8	36.2
Comprehensive	2.2	4.6	2.0	51.5	43.8	55.7
Other	20.9	20.0	30.0	7.5	6.4	8.0
Previous vaccinations, %						
Influenza	29.8	33.2	33.9	35.5	33.3	33.1
Diphtheria, tetanus, and/or pertussis	24.1	26.1	20.0	16.1	15.7	11.3
Pneumonia	4.1	6.8	4.5	10.1	8.8	8.8
Zoster	2.1	1.7	1.7	0.0	0.1	0.2

Abbreviations: NA, not applicable; SD, standard deviation; PPO, preferred provider organization, HMO: health maintenance organization.

* Includes consumer-driven health plan, exclusive provider organization, high deductible health plan, point-of-service, and point-of-service with capitation.

† This vaccination category includes the individual diphtheria vaccine, the individual tetanus vaccine, the combined diphtheria and tetanus vaccine, and the combined diphtheria, tetanus, and pertussis vaccine.

**Figure 1.**
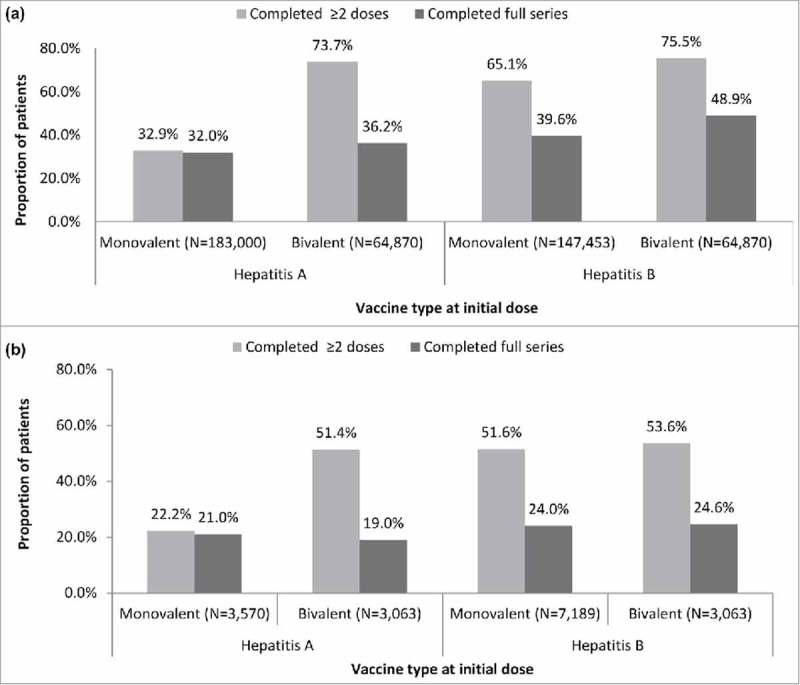
Rates of vaccination series completion among (a) adult commercial/Medicare (N = 350,240) and (b) adult Medicaid patients (N = 12,599)* *Schedule of vaccine series was determined by type of vaccine (monovalent hepatitis A or B or bivalent hepatitis A/B) administered on index date. Completion schedules with a bivalent vaccine on the index date are shown stratified by whether series fulfilled hepatitis A or B coverage; Hepatitis A vaccine is a 2-dose series; however, discrepancies in series completion for hepatitis A allow for bivalent vaccine to be included in the measurement, thus needing three doses, as appropriate, to be complete. The bivalent inclusion required 3 doses for completion.

As shown in Figure 1a, among commercial/Medicare patients who initiated with a monovalent initial dose, 32.0% and 39.6% of patients completed the full HepA and HepB vaccination series, respectively. With a bivalent initial dose, 36.2% and 48.9% of patients completed the full HepA and HepB vaccination series, respectively. Among patients who were initiated on a HepB series, 75.5% received ≥2 doses when the initial dose was with the bivalent vaccine and 65.1% when the initial dose was with a monovalent vaccine.

Among Medicaid patients (Figure 1b), full series completion rates were similar regardless of whether the initial dose was a monovalent or a bivalent vaccine in both the HepA and HepB groups and ranged from 19.0% to 24.6%. Among patients who were initiated on a HepB series, 53.6% received ≥2 doses of HepB vaccination when the initial dose was a bivalent vaccine and 51.6% when the initial dose was a monovalent vaccine.

## Discussion

Assessing HepA and HepB vaccination series completion rates in a large sample from a US insurance claims dataset, we found that 32–36% of commercial/Medicare insurance enrollees received the full HepA vaccination series, and 40–49% received the full HepB vaccination series. The rate varied depending on whether they initiated on a monovalent or bivalent vaccine. Between 65% and 76% of patients initiating HepB vaccination received ≥2 doses. Although this analysis was not designed to compare completion rates between commercial/Medicare and Medicaid enrollees, the respective ranges for Medicaid enrollees were observed to be lower: 19–21% and 24–25% completed a full HepA and HepB vaccine series, respectively, and 52–54% received ≥2 HepB doses. In general, Medicaid enrollees face challenges in terms of gaps in access to certain providers because of provider shortages in low-income communities, lack of transportation, and lower physician fees and participation in Medicaid compared to commercial insurance.^19^

This study updates previously published material on HepA and HepB vaccination series completion rates among adults in the US, which are limited to only one full publication and two announcements in 2017 conferences. Nelson et al. report the results of a retrospective population-based study of patient vaccination data recorded from 1996 to 2004 in the administrative databases of seven health care organizations participating in the Vaccine Safety Datalink Project.^14^ It was found that within one year of the first dose in adults, completion rates ranged 25–44% for HepA and 41–62% for HepB according to the age group (18-29; 30–49; 50–64; ≥ 65 years old).^14^ Their results also corroborate an observation of lower completion rates among Medicaid enrollees in the current study. The latest findings presented at 2017 congresses were derived from the same database as in our study.^20^^,^^21^ They showed that 27%, 51%^20^ and 40%^21^ of the patients received ≥2 doses of HepA, HepB, or HepA-HepB vaccines, respectively. No differentiation, however, was made between Medicaid and commercially insured individuals^20^ who have different socioeconomic, disease and disability status that cannot be controlled for in this database^22^; nor did the definition of series completion account for switching between the bivalent and monovalent hepatitis vaccines.^21^

Finally, a number of studies^13^^,^^23–27^ used the National Health Interview Surveys (NHIS) to assess hepatitis vaccination coverage but none examined the series completion rates neither the type of vaccine (monovalent or bivalent) administered.

Potential barriers to vaccination series completion may include administrative or financial barriers, limited access to vaccination services, or lack of awareness of recommended schedules among patients. For example, travelers who were partially vaccinated with HAV and HBV, believed that they had received the recommended number of HepA and HepB vaccine doses.^17^ In another study, medical visits prior to or after starting HepA or HepB vaccination series was found to facilitate series completion.^14^^,^^28^

Our results highlight the need for future research to determine the effectiveness of interventions to increase vaccination completion rates. Electronic health records with complete information on patient's immunization history are among the Standards for Adult Immunization Practice recommended by the National Vaccine Advisory Committee.^29^ Previous investigations have shown that electronic medical record (EMR) reminders significantly improved the rates of influenza and pneumococcal vaccination in a population of patients over 65 years of age.^30^ More recent analyses^31^^,^^32^ compared 3-dose HepB vaccine completion rates in patients with diabetes aged 19–59 years between two large US managed care organizations, Kaiser Permanente Southern California in which an electronic alert and a reminder were implemented in the EMR and Kaiser Permanente Northern California in which no such reminder was used.^31^ The site with EMR reminders increased vaccination coverage rapidly from 7.6% in 2012 to 29.4% in 2015, while no increase was seen at the site with no EMR reminder.^31^^,^^32^ EMR are increasingly being adopted by health systems,^31^ and might therefore be a valuable tool for improving vaccination completion.

### Limitations

Selection of patients with a HepA, HepB, or HepA-HepB vaccine was based on CPT codes, and coding inaccuracies may lead to misclassification bias and misidentification of patients with a hepatitis vaccine. In addition, CPT codes cannot distinguish between brands of hepatitis vaccines and thus cannot differentiate between vaccination schedules for the two HepA vaccines. Completion windows were adjusted to enable completion of the longest time window within each vaccine series. Finally, our population was covered by employer-sponsored health care with supplemental Medicare plans, or Medicaid, and may not be generalizable to an uninsured population.

## Methods

### Study design

We used the Truven Health Analytics MarketScan claims database, which is a large US administrative claims database. Data retrieved included Q1 2007-Q3 2015 data from the Commercial Claims and Encounters database and the Medicare Supplemental and Coordination of Benefits database (“commercial/Medicare” cohort) and Q1 2007-Q4 2014 data from the Medicaid Multi-State database (“Medicaid” cohort). The Truven MarketScan database is de-identified and complies with the Health Insurance Portability and Accountability Act (HIPAA) to preserve patient anonymity and confidentiality.

**Figure 2.**
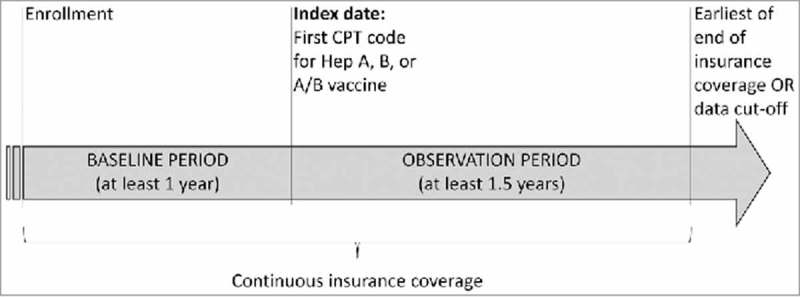
Study design scheme; Abbreviations: CPT, Current Procedural Terminology *Hepatitis A adult dosage, CPT 90632; Hepatitis B adult standard 3 doses, CPT: 90740; Hepatitis B for dialysis or immunocompromised patients, 3 doses, CPT: 90746; Hepatitis B for dialysis or immunocompromised patients, 4 doses, CPT: 90747; Hepatitis A and B adult dosage, CPT: 90636.

The date of the first Current Procedural Terminology (CPT) code for a HepA, HepB, or HepA-HepB vaccine was defined as the ‘index date’. More precisely, patients who initiated a series for HepA and another series for HepB vaccine with monovalent vaccines had distinct index dates that corresponded to the first dose of each vaccine series. However, once a patient had initiated a HepA or HepB series, their index date for each series was locked. Thus, patients who initiated with a monovalent vaccine could not have a later bivalent index date, and patients who initiated with a bivalent vaccine could not have a later monovalent index date. Identification of subsequent doses was also based on CPT codes. Study periods consisted of a ‘baseline period’ that was the period from the date of health plan enrollment up to and including the index date, and an ‘observation period’ that was the period from the index date to the end of insurance coverage or data cut-off (Figure 2).

### Study population

Patients were included in the study if they had ≥1 claim with a CPT code for HepA, HepB, or HepA-HepB vaccine, were ≥19 years of age at index date, and had continuous insurance enrollment for ≥12 months before the index date (“baseline period”) and ≥18 months after the index date (“observation period”). This was to allow for the evaluation of vaccination series completion, because the minimum required time for the completion of a full series vaccination against HepA is 6 months and against HepB is 4 months (Table 2). Patients were excluded if they had ≥2 claims with HepA diagnoses before HepA or HepA-HepB index dates or ≥2 claims with HepB diagnoses before HepB orHepA-HepB index date.

**Table 2.** Hepatitis A, B, or A/B Vaccine Completion Adult Schedule Guidelines^11^^,^^12^

Type of Vaccine^,^	Dosing Schedule	Completion criteria
Monovalent HepA	2-dose series given on a 0-, 6–12 or a 0-, 6-18-month schedule depending on the HAV vaccine	Time b/w 1^st^ and 2^nd^ dose ≥6 months
Monovalent HepB	3-dose series given on a 0-, 1-, 6-month schedule4-dose series given on a 0-, 1-, 2-, 6-month schedule for adults on hemodialysis	Time b/w 1^st^ and 2^nd^ dose is ≥1 monthTime b/w 2^nd^ and 3^rd^ dose is ≥8 weeksTime b/w 1^st^ and 3^rd^ dose is ≥16 weeksTime b/w 1^st^ and 2^nd^ dose is ≥1 monthTime b/w 2^nd^ and 3^rd^ dose is ≥4 weeksTime b/w 1^st^ and 4^th^ dose is ≥16 weeks
Bivalent HepA-HepB	Standard dosing: 3-dose series given on a 0-, 1-, and 6-month schedule Accelerated dosing: 4-dose series given on days 0, 7, and 21 to 30 followed by a booster dose at month 12	HAV series completion criteria:Time b/w 1^st^ and 2^nd^ dose is ≥1 monthTime b/w 2^nd^ and 3^rd^ dose is ≥5 monthsHBV series completion criteria:Time b/w 1^st^ and 2^nd^ dose is ≥1 monthTime b/w 2^nd^ and 3^rd^ dose is ≥8 weeksTime b/w 1^st^ and 3^rd^ dose is ≥16 weeksNA

a Type of vaccine (monovalent HepA or HepB or bivalent HepA-HepB) administered on index date was used to determine schedule of vaccine series.

b Vaccine series may include: 1) monovalent vaccines only, 2) a combination of both monovalent and bivalent vaccines, 3) either monovalent vaccines only or a combination of monovalent and bivalent vaccines, 4) bivalent vaccines only, or 5) either bivalent vaccines only or a combination of monovalent and bivalent vaccines.

c Completion schedules when initiating with a bivalent vaccine on the index date were stratified by whether series fulfilled hepatitis A or B coverage.

d Because CPT codes do not differentiate between standard dosing and accelerate dosing vaccination schedules, completion was assessed as at least 3 doses as per the standard dosing schedule.

### Outcomes

HepA and HepB vaccine series completion was assessed separately for HepA and HepB and was defined as a series of either monovalent HepA or HepB vaccines, bivalent vaccines, or a combination of monovalent HepA or HepB vaccines with bivalent HepA-HepB vaccine, as shown in Table 2. In addition, only doses separated by minimum intervals based on schedules per CDC guidelines were included, and identified using CPT codes in the datasets^11^^,^^12^ (Table 2). The proportion of patients in each cohort who received ≥2 doses of HepB vaccination was also assessed.

### Statistical analyses

All analyses were stratified by commercial/Medicare and Medicaid patient cohorts. Descriptive statistics were generated to summarize patient baseline characteristics and series completion. Additionally, the number and proportion of patients completing ≥2 doses of HepB and the full HepA and HepB series were assessed and analyses were stratified according to whether the initial dose was a monovalent or bivalent vaccine.

## Conclusions

HepA and HepB vaccination series completion rates were low among US adults, and appeared to be generally lower among Medicaid than among commercial/Medicare enrollees. Adult commercial/Medicare patients starting hepatitis vaccination series with the bivalent vaccine had higher rates of completion than those starting with monovalent vaccines – an observation that was not made among Medicaid enrollees. Low rates of HepA and HepB vaccination series completion rates among adults in the US, demonstrate the need for interventions that increase series completion, thereby offering optimal protection for individual patients, and contributing to public health.

## Clinical trial registration

Not applicable.

## Prior presentation

ISPOR 22^nd^ Annual International Meeting; May 20–24, 2017; Boston, MA, USA.

## Abbreviations

ACIP: Advisory Committee on Immunization Practices
CI: confidence interval
CDC: Centers for Disease Control and Prevention
CPT: Current Procedural Terminology
EMR: electronic medical record
HAV: hepatitis A virus
HBV: hepatitis B virus
HepA: hepatitis A
HepB: hepatitis B
US: United States
